# Supplementary material for: Possible Event-Related Potential Correlates of Voluntary Attention and Reflexive Attention in the Emei Music Frog
Source: Biology (Basel). 2022 Jun 8;11(6):879. doi: 10.3390/biology11060879 (PMC9219635; doi:10.3390/biology11060879)
Supplement: Supplementary file 1 [file biology-11-00879-s001.zip › biology-1611729-supplementary.pdf]

Possible event-related potential correlates of voluntary attention and reflexive attention in the Emei music frog

Journal name: *Biology*

Wenjun Niu[1, 2] Di Shen[1, 3] Ruolei Sun[2] Yanzhu Fan[1, 3] Jing Yang[1, 3] Baowei Zhang[2] and Guangzhan Fang[1, 3 *]

[1] Chengdu Institute of Biology, Chinese Academy of Sciences, Chengdu 610041, Sichuan, China

[2] School of Life Science, Anhui University, Hefei 230601, Anhui, China

[3] University of Chinese Academy of Sciences, 19A Yuquan Road, Beijing 100049, China

[*] Authors for correspondence: Guangzhan Fang, e-mail: fanggz@cib.ac.cn

Address: Chengdu Institute of Biology, Chinese Academy of Sciences

Tel: +86-28-82890628

Supplementary materials

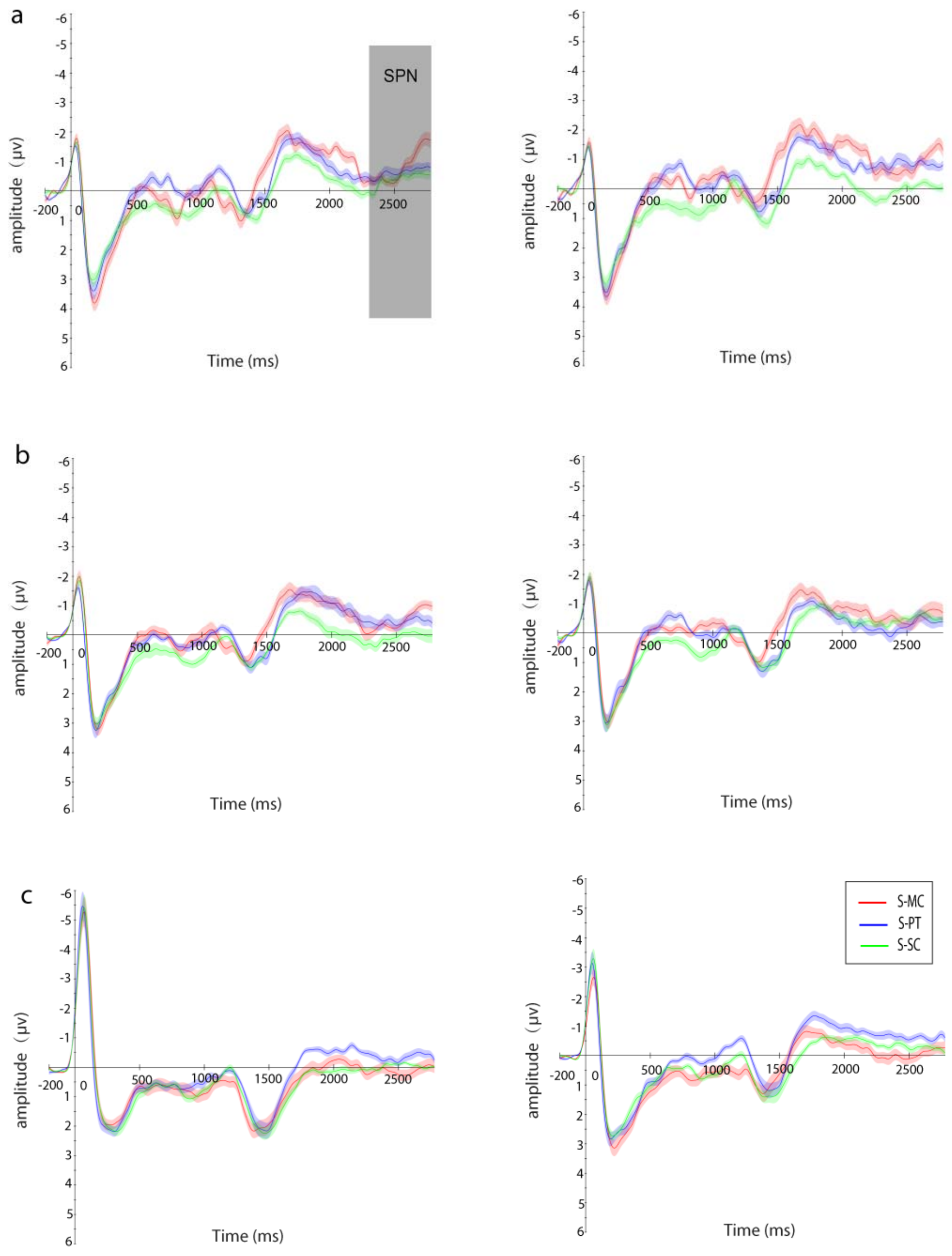

Figure S1. Grand average waveforms with half of the standard errors for the experiments recruiting voluntary attention for the left and right telencephalon (a), diencephalon (b) and mesencephalon (c). SPN was acquired from superimposition of the recorded waveforms according to the onset of white noise before silence replacement. S-MC, using silence replaced male calls; S-PT, using silence replaced pure tone; S-SC, using silence replaced screech calls.

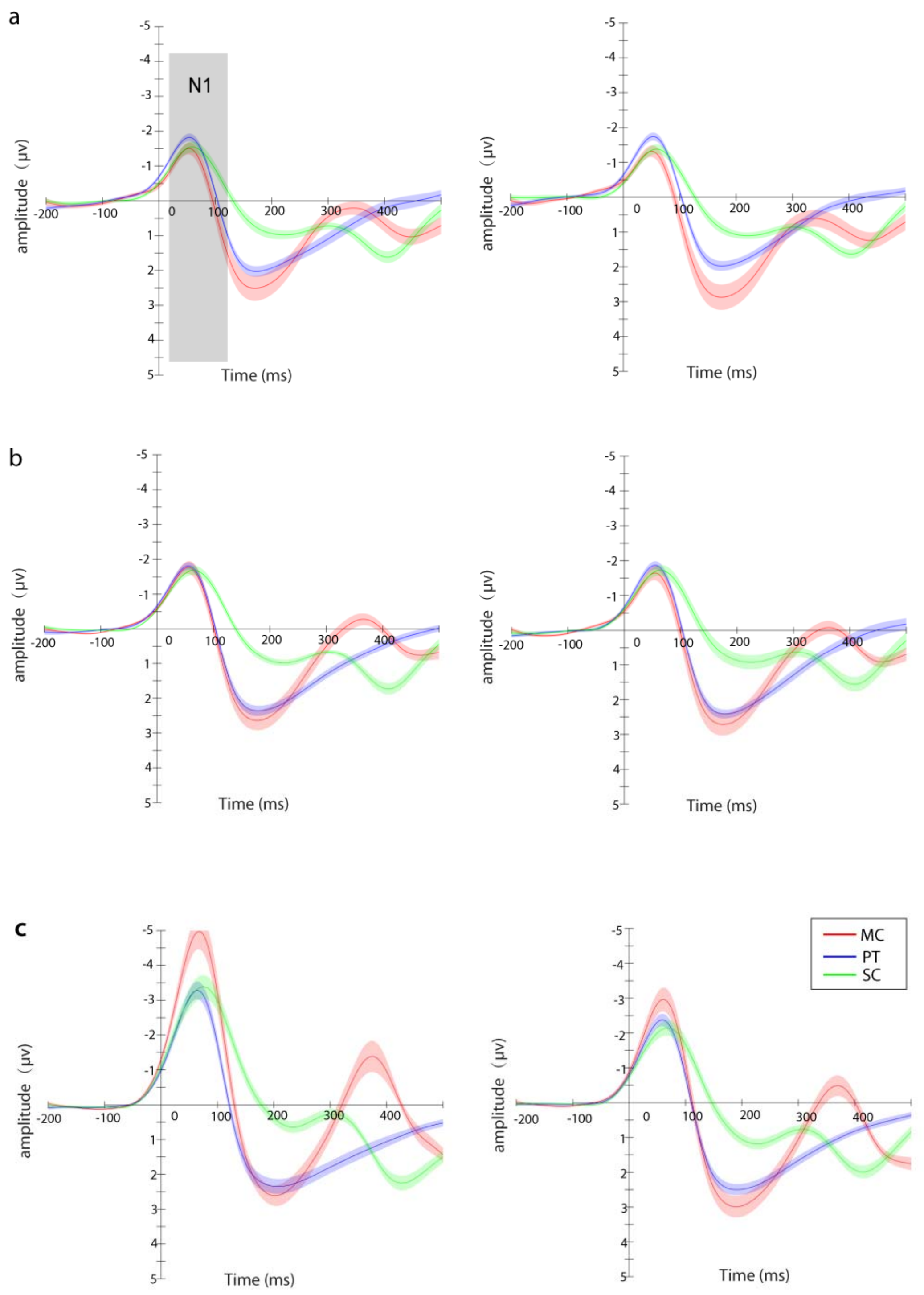

Figure S2. Grand average waveforms with half of the standard errors evoked by conspecific calls, pure tone and screech calls in the left and right telencephalon (a), diencephalon (b) and mesencephalon (c) for the experiments recruiting voluntary attention. The ERP components were acquired from superimposition of the recorded waveforms according to the onset of the target stimuli. MC, conspecific male advertisement call; PT, 1000 Hz pure tone; SC, screech call.

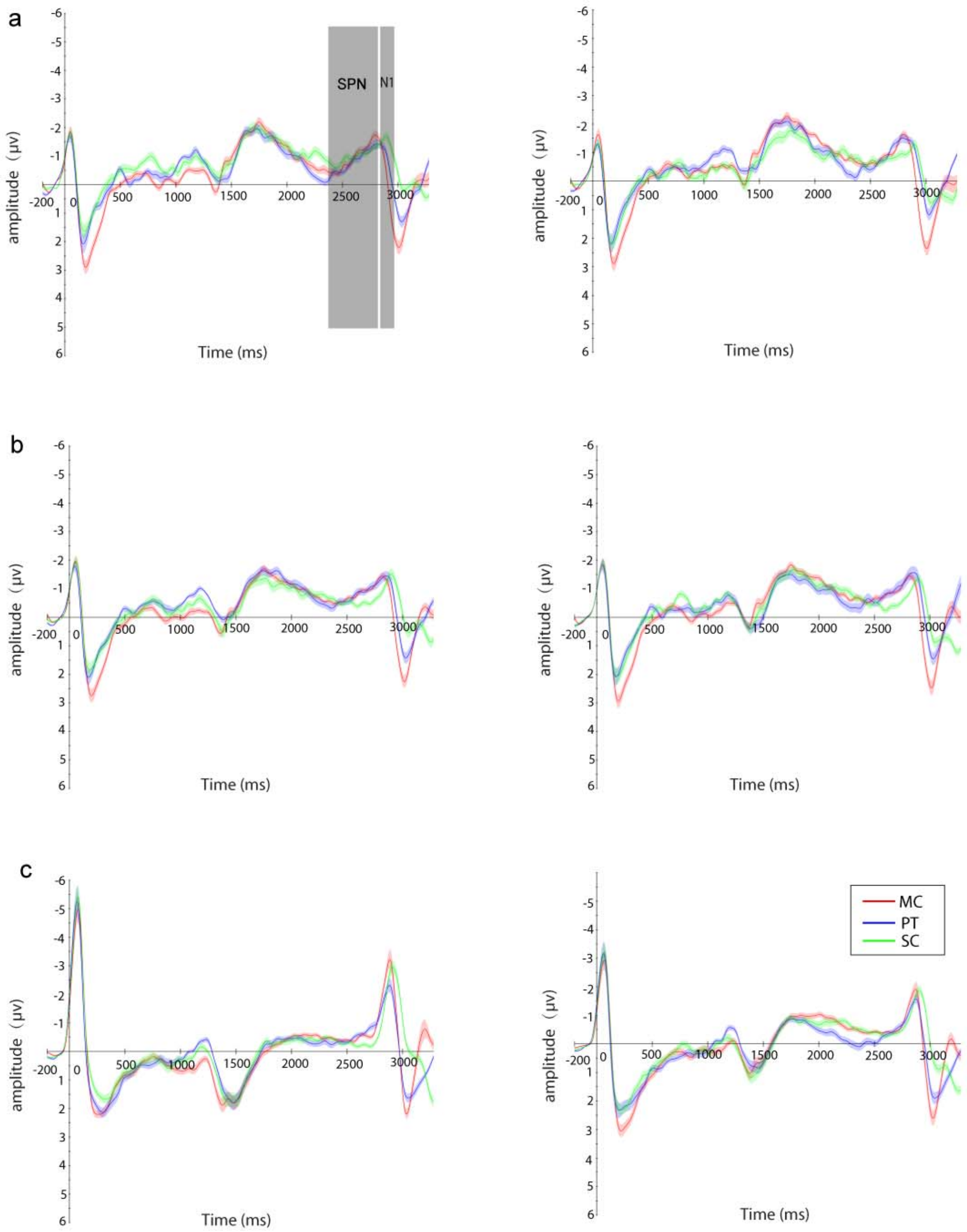

Figure S3. Grand average waveforms with half of the standard errors by conspecific calls, pure tone and screech calls in the left and right telencephalon (a), diencephalon (b), and mesencephalon (c) for the experiments recruiting voluntary attention. The ERP components were acquired from superimposition of the recorded waveforms according to the onset of white noise before the target stimuli. MC, conspecific male advertisement call; PT, 1000 Hz pure tone; SC, screech call.

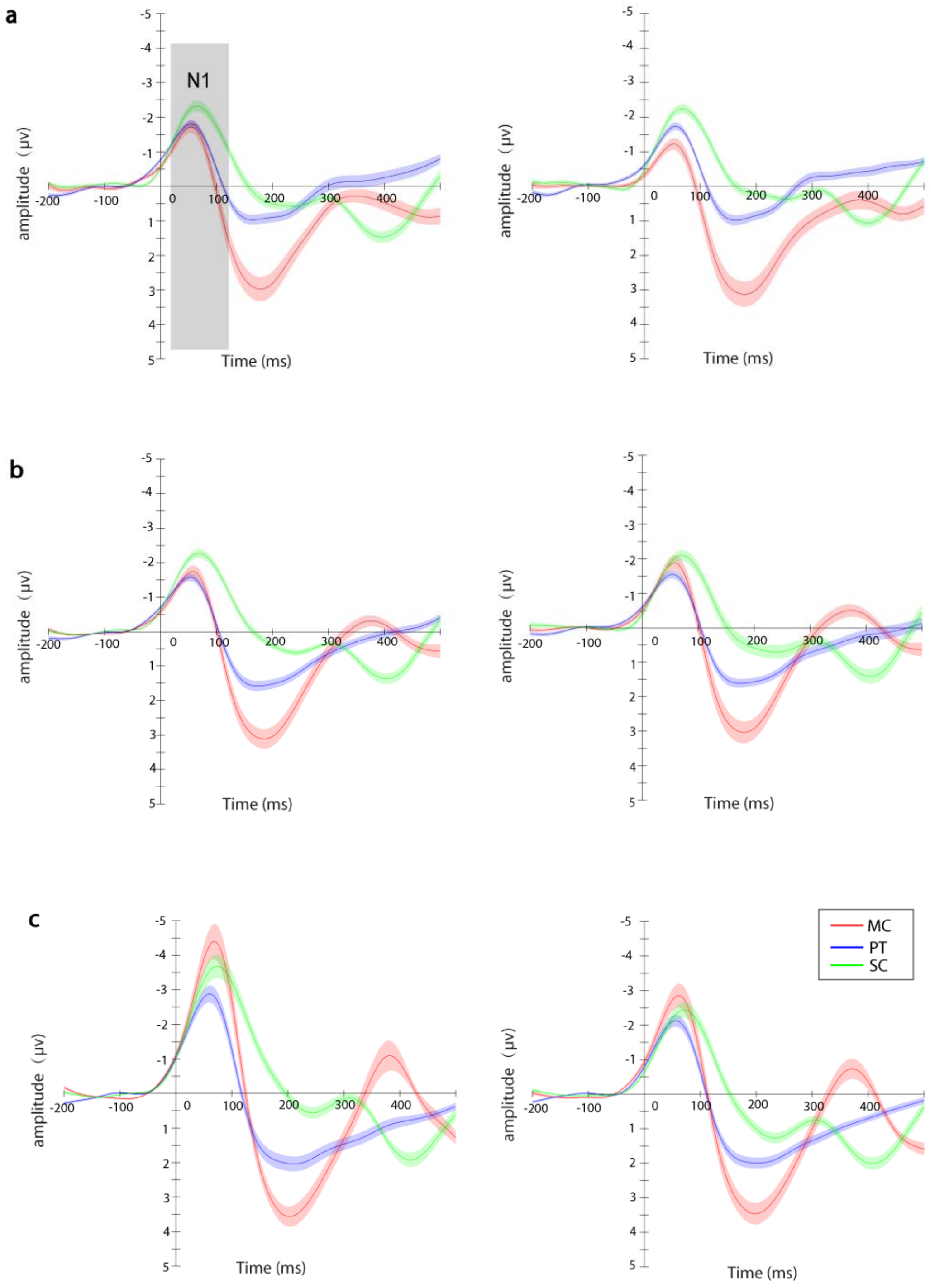

Figure S4. Grand average waveforms with half of the standard errors evoked by conspecific calls, pure tone and screech calls in the left and right telencephalon (a), diencephalon (b) and mesencephalon (c) for the experiments recruiting reflexive attention. MC, conspecific male advertisement call; PT, 1000 Hz pure tone; SC, screech call.
